# Supplementary material for: Critical evaluation of kinetic schemes for coagulation
Source: PLoS One. 2023 Aug 28;18(8):e0290531. doi: 10.1371/journal.pone.0290531 (PMC10461854; doi:10.1371/journal.pone.0290531)
Supplement: S1 Text — (PDF) [file pone.0290531.s001.pdf]

## Supporting Information

**Table 1. Original Int Model from Chatterjee et al.**

| # | Reaction | $k_1$ | $k_{-1}$ | $k_{cat}$ |
|---|---|---|---|---|
| 1 | TF + VII ↔ TF = VII | $3.2x10^6$ $M^{-1}s^{-1}$ | $3.1x10^{-3}$ $s^{-1}$ | |
| 2 | TF + VIIa ↔ TF = VIIa | $2.3x10^7$ $M^{-1}s^{-1}$ | $3.1x10^{-3}$ $s^{-1}$ | |
| 3 | TF = VIIa + VII → TF = VIIa + VIIa | $4.4x10^5$ $M^{-1}s^{-1}$ | | |
| 4 | Xa + VII → Xa + VIIa | $1.3x10^7$ $M^{-1}s^{-1}$ | | |
| 5 | IIa + VII → IIa + VIIa | $2.3x10^4$ $M^{-1}s^{-1}$ | | |
| 6 | TF = VIIa + X ↔ TF = VIIa = X → TF = VIIa = Xa | $2.5x10^7$ $M^{-1}s^{-1}$ | 1.05 $s^{-1}$ | 6 $s^{-1}$ |
| 7 | TF = VIIa + Xa ↔ TF = VIIa = Xa | $2.2x10^7$ $M^{-1}s^{-1}$ | 19 $s^{-1}$ | |
| 8 | TF = VIIa + IX ↔ TF = VIIa = IX → TF = VIIa + IXa | $1.0x10^7$ $M^{-1}s^{-1}$ | 2.4 $s^{-1}$ | 1.8 $s^{-1}$ |
| 9 | II + Xa → IIa + Xa | $7.5x10^3$ $M^{-1}s^{-1}$ | | |
| 10 | IIa + VIII → IIa + VIIIa | $2.0x10^7$ $M^{-1}s^{-1}$ | | |
| 11 | VIIIa + IXa ↔ IXa = VIIIa | $1.0x10^7$ $M^{-1}s^{-1}$ | $5.0x10^{-3}$ $s^{-1}$ | |
| 12 | IXa = VIIIa + X ↔ IXa = VIIIa = X → IXa = VIIIa + Xa | $1.0x10^8$ $M^{-1}s^{-1}$ | $1.0x10^{-3}$ $s^{-1}$ | 8.2 $s^{-1}$ |
| 13 | VIIIa ↔ $VIIIa_1$ + $VIIIa_2$ | $6.0x10^{-3}$ $s^{-1}$ | $2.2x10^4$ $M^{-1}s^{-1}$ | |
| 14 | IXa = VIIIa = X → IXa + X + $VIIIa_1$ + $VIIIa_2$ | $1.0x10^{-3}$ $s^{-1}$ | | |
| 15 | IXa = VIIIa → IXa + $VIIIa_1$ + $VIIIa_2$ | $1.0x10^{-3}$ $s^{-1}$ | | |
| 16 | IIa + V → IIa + Va | $2.0x10^7$ $M^{-1}s^{-1}$ | | |
| 17 | Xa + Va ↔ Xa = Va | $4.0x10^8$ $M^{-1}s^{-1}$ | 0.2 $s^{-1}$ | |
| 18 | Xa = Va + II ↔ Xa = Va = II → Xa = Va + mIIa | $1.0x10^8$ $M^{-1}s^{-1}$ | 103 $s^{-1}$ | 63.5 $s^{-1}$ |
| 19 | Xa = Va + mIIa → Xa = Va + IIa | $1.5x10^7$ $M^{-1}s^{-1}$ | | |
| 20 | Xa + TFPI ↔ Xa = TFPI | $9.0x10^5$ $M^{-1}s^{-1}$ | $3.6x10^{-4}$ $s^{-1}$ | |
| 21 | TF = VIIa = Xa + TFPI ↔ TF = VIIa = Xa = TFPI | $3.2x10^8$ $M^{-1}s^{-1}$ | $1.1x10^{-4}$ $s^{-1}$ | |
| 22 | TF = VIIa + Xa = TFPI → TF = VIIa = Xa + TFPI | $5.0x10^7$ $M^{-1}s^{-1}$ | | |
| 23 | Xa + ATIII → Xa = ATIII | $1.5x10^3$ $M^{-1}s^{-1}$ | | |
| 24 | mIIa + ATIII → mIIa = ATIII | $7.1x10^3$ $M^{-1}s^{-1}$ | | |
| 25 | IXa + ATIII → IXa = ATIII | $4.9x10^2$ $M^{-1}s^{-1}$ | | |
| 26 | IIa + ATIII → IIa = ATIII | $7.1x10^3$ $M^{-1}s^{-1}$ | | |
| 27 | TF = VIIa + ATIII → TF = VIIa = ATIII | $2.3x10^2$ $M^{-1}s^{-1}$ | | |
| 28 | Boc-VPR-MCA + IIa ↔ Boc-VPR-MCA = IIa → Boc-VPR + MCA + IIa | $1.0x10^8$ $M^{-1}s^{-1}$ | $6.1x10^3$ $s^{-1}$ | 53.8 $s^{-1}$ |
| 29 | XII → XIIa | $5.0x10^{-4}$ $s^{-1}$ | | $3.3x10^{-2}$ $s^{-1}$ |
| 30 | XIIa + XII ↔ XIIa = XII → XIIa + XIIa | $1.0x10^8$ $M^{-1}s^{-1}$ | 750 $s^{-1}$ | 40 $s^{-1}$ |
| 31 | XIIa + PK ↔ XIIa = PK → XIIa + K | $1.0x10^8$ $M^{-1}s^{-1}$ | $3.6x10^3$ $s^{-1}$ | 5.7 $s^{-1}$ |
| 32 | XII + K ↔ XII = K → XIIa + K | $1.0x10^8$ $M^{-1}s^{-1}$ | 45.3 $s^{-1}$ | |
| 33 | PK + K → K + K | $2.7x10^4$ $M^{-1}s^{-1}$ | | |
| 34 | K → K.inhibited | $1.1x10^{-2}$ $s^{-1}$ | | |
| 35 | XIIa + CTI ↔ XIIa = CTI | $1.0x10^8$ $M^{-1}s^{-1}$ | 2.4 $s^{-1}$ | |
| 36 | XIIa + C1inh → XIIa = C1inh | $3.6x10^3$ $M^{-1}s^{-1}$ | | |
| 37 | XIIa + ATIII → XIIa = ATIII | 21.6 $M^{-1}s^{-1}$ | | |
| 38 | XI + IIa ↔ XI = IIa → XIa + IIa | $1.0x10^8$ $M^{-1}s^{-1}$ | 5 $s^{-1}$ | $1.3x10^{-4}$ $s^{-1}$ |
| 39 | XIIa + XI ↔ XIIa = XI → XIIa + XIa | $1.0x10^8$ $M^{-1}s^{-1}$ | 200 $s^{-1}$ | $5.7x10^{-4}$ $s^{-1}$ |
| 40 | XIa + XI ↔ XIa = XI→ XIa + XIa | $3.19x10^6$ $M^{-1}s^{-1}$ | | |
| 41 | XIa + ATIII → XIa = ATIII | $3.2x10^2$ $M^{-1}s^{-1}$ | | |
| 42 | XIa + C1inh → XIa = C1inh | $1.8x10^3$ $M^{-1}s^{-1}$ | | |
| 43 | XIa + α1AT → XIa = α1AT | $1.0x10^2$ $M^{-1}s^{-1}$ | | |
| 44 | XIa + α2AP → XIa = α2AP | $4.3x10^3$ $M^{-1}s^{-1}$ | | |
| 45 | XIa + IX ↔ XIa = IX → XIa + IXa | $1.0x10^8$ $M^{-1}s^{-1}$ | 41 $s^{-1}$ | 7.7 $s^{-1}$ |
| 46 | IXa + X ↔ IXa = X → IXa + Xa | $1.0x10^8$ $M^{-1}s^{-1}$ | 0.64 $s^{-1}$ | $7.0x10^{-4}$ $s^{-1}$ |
| 47 | Xa + VIII ↔ Xa = VIII → Xa + VIIIa | $1.0x10^8$ $M^{-1}s^{-1}$ | 20.1 $s^{-1}$ | 0.023 $s^{-1}$ |
| 48 | VIIa + IX ↔ VIIa = IX → VIIa + IXa | $1.0x10^8$ $M^{-1}s^{-1}$ | 0.9 $s^{-1}$ | $3.6x10^{-5}$ $s^{-1}$ |
| 49 | VIIa + X ↔ VIIa = X → VIIa + Xa | $1.0x10^8$ $M^{-1}s^{-1}$ | 210 $s^{-1}$ | $1.6x10^{-6}$ $s^{-1}$ |
| 50 | Fbg + IIa ↔ Fbg = IIa → Fbn1 + IIa + FPA | $1.0x10^8$ $M^{-1}s^{-1}$ | 636 $s^{-1}$ | 84 $s^{-1}$ |

| | | | | |
|---|---|---|---|---|
| 51 | Fbn1 + IIa ↔ Fbn1 = IIa → Fbn2 + IIa + FPB | $1.0x10^{8}$ $M^{-1}s^{-1}$ | 742.6 $s^{-1}$ | 7.4 $s^{-1}$ |
| 52 | 2Fbn1 ↔ $(Fbn1)_2$ | $1.0x10^{6}$ $M^{-1}s^{-1}$ | 6.4 $x10^{-2}$ $s^{-1}$ | |
| 53 | $(Fbn1)_2$ + IIa ↔ $(Fbn1)_2$ = IIa → $(Fbn2)_2$ + IIa + FPB | $1.0x10^{8}$ $M^{-1}s^{-1}$ | 701 $s^{-1}$ | 49 $s^{-1}$ |
| 54 | Fbn2 + IIa ↔ Fbn2 = IIa | $1.0x10^{8}$ $M^{-1}s^{-1}$ | 1.0 $x10^{3}$ $s^{-1}$ | |
| 55 | $(Fbn1)_2$ = IIa + ATIII → $(Fbn1)_2$ = IIa = ATIII | $1.6x10^{4}$ $M^{-1}s^{-1}$ | | |
| 56 | Fbn1 = IIa + ATIII → Fbn1 = IIa = ATIII | $1.6x10^{4}$ $M^{-1}s^{-1}$ | | |
| 57 | Fbn2 = IIa + ATIII → Fbn2 = IIa = ATIII | $1.6x10^{4}$ $M^{-1}s^{-1}$ | | |

**Table 2. Original Ext Model from Butenas et al.**

| # | Reaction | $k_1$ | $k_{-1}$ | $k_{cat}$ |
|---|---|---|---|---|

| | | | | |
|---|---|---|---|---|
| 1 | TF + VII ↔ TF = VII | $3.2x10^{6}$ $M^{-1}s^{-1}$ | $3.1x10^{-3}$ $s^{-1}$ | |
| 2 | TF + VIIa ↔ TF = VIIa | $2.3x10^{7}$ $M^{-1}s^{-1}$ | $3.1x10^{-3}$ $s^{-1}$ | |
| 3 | TF = VIIa + VII ⟶ TF = VIIa + VIIa | $4.4x10^{5}$ $M^{-1}s^{-1}$ | | |
| 4 | Xa + VII ⟶ Xa + VIIa | $1.3x10^{7}$ $M^{-1}s^{-1}$ | | |
| 5 | IIa + VII ⟶ IIa + VIIa | $2.3x10^{4}$ $M^{-1}s^{-1}$ | | |
| 6 | TF = VIIa + X ↔ TF = VIIa = X ⟶ TF = VIIa = Xa | $2.5x10^{7}$ $M^{-1}s^{-1}$ | 1.05 $s^{-1}$ | 6 $s^{-1}$ |
| 7 | TF = VIIa + Xa ↔ TF = VIIa = Xa | $2.2x10^{7}$ $M^{-1}s^{-1}$ | 19 $s^{-1}$ | |
| 8 | TF = VIIa + IX ↔ TF = VIIa = IX ⟶ TF = VIIa + IXa | $1.0x10^{7}$ $M^{-1}s^{-1}$ | 2.4 $s^{-1}$ | 1.8 $s^{-1}$ |
| 9 | II + Xa ⟶ IIa + Xa | $7.5x10^{3}$ $M^{-1}s^{-1}$ | | |
| 10 | IIa + VIII ⟶ IIa + VIIIa | $2.0x10^{7}$ $M^{-1}s^{-1}$ | | |
| 11 | VIIIa + IXa ↔ IXa = VIIIa | $1.0x10^{7}$ $M^{-1}s^{-1}$ | $5.0x10^{-3}$ $s^{-1}$ | |
| 12 | IXa = VIIIa + X ↔ IXa = VIIIa = X ⟶ IXa = VIIIa + Xa | $1.0x10^{8}$ $M^{-1}s^{-1}$ | $1.0x10^{-3}$ $s^{-1}$ | 8.2 $s^{-1}$ |
| 13 | VIIIa ↔ $VIIIa_1$ + $VIIIa_2$ | $6.0x10^{-3}$ $s^{-1}$ | $2.2x10^{4}$ $M^{-1}s^{-1}$ | |
| 14 | IXa = VIIIa = X ⟶ IXa + X + $VIIIa_1$ + $VIIIa_2$ | $1.0x10^{-3}$ $s^{-1}$ | | |
| 15 | IXa = VIIIa ⟶ IXa + $VIIIa_1$ + $VIIIa_2$ | $1.0x10^{-3}$ $s^{-1}$ | | |
| 16 | IIa + V ⟶ IIa + Va | $2.0x10^{7}$ $M^{-1}s^{-1}$ | | |
| 17 | Xa + Va ↔ Xa = Va | $4.0x10^{8}$ $M^{-1}s^{-1}$ | 0.2 $s^{-1}$ | |
| 18 | Xa = Va + II ↔ Xa = Va = II ⟶ Xa = Va + mIIa | $1.0x10^{8}$ $M^{-1}s^{-1}$ | 103 $s^{-1}$ | 63.5 $s^{-1}$ |
| 19 | Xa = Va + mIIa ⟶ Xa = Va + IIa | $1.5x10^{7}$ $M^{-1}s^{-1}$ | | |
| 20 | Xa + TFPI ↔ Xa = TFPI | $9.0x10^{5}$ $M^{-1}s^{-1}$ | $3.6x10^{-4}$ $s^{-1}$ | |
| 21 | TF = VIIa = Xa + TFPI ↔ TF = VIIa = Xa = TFPI | $3.2x10^{8}$ $M^{-1}s^{-1}$ | $1.1x10^{-4}$ $s^{-1}$ | |
| 22 | TF = VIIa + Xa = TFPI ⟶ TF = VIIa = Xa + TFPI | $5.0x10^{7}$ $M^{-1}s^{-1}$ | | |
| 23 | Xa + ATIII ⟶ Xa = ATIII | $1.5x10^{3}$ $M^{-1}s^{-1}$ | | |
| 24 | mIIa + ATIII ⟶ mIIa = ATIII | $7.1x10^{3}$ $M^{-1}s^{-1}$ | | |
| 25 | IXa + ATIII ⟶ IXa = ATIII | $4.9x10^{2}$ $M^{-1}s^{-1}$ | | |
| 26 | IIa + ATIII ⟶ IIa = ATIII | $7.1x10^{3}$ $M^{-1}s^{-1}$ | | |
| 27 | TF = VIIa + ATIII ⟶ TF = VIIa = ATIII | $2.3x10^{2}$ $M^{-1}s^{-1}$ | | |
| 28 | IXa + X ⟶ IXa + Xa | $K_m$ = $1,4x10^{-7}$ M | $K_{cat}$ = $8x10^{-4}$ $s^{-1}$ | |

**Table 3. Modified Int Model**

| # | Reaction | $k_1$ | $k_{-1}$ | $k_{cat}$ |
|---|---|---|---|---|

| | | | | |
|---|---|---|---|---|
| 1 | TF + VII ↔ TF = VII | 3.2x10$^{6}$ M$^{-1}$s$^{-1}$ | 3.1x10$^{-3}$ s$^{-1}$ | |
| 2 | TF + VIIa ↔ TF = VIIa | 2.3x10$^{7}$ M$^{-1}$s$^{-1}$ | 3.1x10$^{-3}$ s$^{-1}$ | |
| 3 | TF = VIIa + VII → TF = VIIa + VIIa | 4.4x10$^{5}$ M$^{-1}$s$^{-1}$ | | |
| 4 | Xa + VII → Xa + VIIa | 1.3x10$^{7}$ M$^{-1}$s$^{-1}$ | | |
| 5 | IIa + VII → IIa + VIIa | 2.3x10$^{4}$ M$^{-1}$s$^{-1}$ | | |
| 6 | TF = VIIa + X ↔ TF = VIIa = X → TF = VIIa = Xa | 2.5x10$^{7}$ M$^{-1}$s$^{-1}$ | 1.05 s$^{-1}$ | 6 s$^{-1}$ |
| 7 | TF = VIIa + Xa ↔ TF = VIIa = Xa | 2.2x10$^{7}$ M$^{-1}$s$^{-1}$ | 19 s$^{-1}$ | |
| 8 | TF = VIIa + IX ↔ TF = VIIa = IX → TF = VIIa + IXa | 1.0x10$^{7}$ M$^{-1}$s$^{-1}$ | 2.4 s$^{-1}$ | 1.8 s$^{-1}$ |
| 9 | II + Xa → IIa + Xa | 7.5x10$^{3}$ M$^{-1}$s$^{-1}$ | | |
| 10 | IIa + VIII → IIa + VIIIa | 2.0x10$^{7}$ M$^{-1}$s$^{-1}$ | | |
| 11 | VIIIa + IXa ↔ IXa = VIIIa | 1.0x10$^{7}$ M$^{-1}$s$^{-1}$ | 5.0x10$^{-3}$ s$^{-1}$ | |
| 12 | IXa = VIIIa + X → IXa = VIIIa + Xa | 0.19x10$^{-6}$ M | | 29 s$^{-1}$ |
| 13 | VIIIa ↔ VIIIa$_1$ + VIIIa$_2$ | 6.0x10$^{-3}$ s$^{-1}$ | 2.2x10$^{4}$ M$^{-1}$s$^{-1}$ | |
| 14 | IXa = VIIIa = X → IXa + X + VIIIa$_1$ + VIIIa$_2$ | 1.0x10$^{-3}$ s$^{-1}$ | | |
| 15 | IXa = VIIIa → IXa + VIIIa$_1$ + VIIIa$_2$ | 1.0x10$^{-3}$ s$^{-1}$ | | |
| 16 | IIa + V → IIa + Va | 2.0x10$^{7}$ M$^{-1}$s$^{-1}$ | | |
| 17 | Xa + Va ↔ Xa = Va | 4.0x10$^{8}$ M$^{-1}$s$^{-1}$ | 0.2 s$^{-1}$ | |
| 18 | Xa = Va + II ↔ Xa = Va = II → Xa = Va + mIIa | 1.0x10$^{8}$ M$^{-1}$s$^{-1}$ | 103 s$^{-1}$ | 63.5 s$^{-1}$ |
| 19 | Xa = Va + mIIa → Xa = Va + IIa | 1.5x10$^{7}$ M$^{-1}$s$^{-1}$ | | |
| 20 | Xa + TFPI ↔ Xa = TFPI | 9.0x10$^{5}$ M$^{-1}$s$^{-1}$ | 3.6x10$^{-4}$ s$^{-1}$ | |
| 21 | TF = VIIa = Xa + TFPI ↔ TF = VIIa = Xa = TFPI | 3.2x10$^{8}$ M$^{-1}$s$^{-1}$ | 1.1x10$^{-4}$ s$^{-1}$ | |
| 22 | TF = VIIa + Xa = TFPI → TF = VIIa = Xa + TFPI | 5.0x10$^{7}$ M$^{-1}$s$^{-1}$ | | |
| 23 | Xa + ATIII → Xa = ATIII | 1.5x10$^{3}$ M$^{-1}$s$^{-1}$ | | |
| 24 | mIIa + ATIII → mIIa = ATIII | 7.1x10$^{3}$ M$^{-1}$s$^{-1}$ | | |
| 25 | IXa + ATIII → IXa = ATIII | 4.9x10$^{2}$ M$^{-1}$s$^{-1}$ | | |
| 26 | IIa + ATIII → IIa = ATIII | 7.1x10$^{3}$ M$^{-1}$s$^{-1}$ | | |
| 27 | TF = VIIa + ATIII → TF = VIIa = ATIII | 2.3x10$^{2}$ M$^{-1}$s$^{-1}$ | | |
| 28 | XII → XIIa | 5.0x10$^{-4}$ s$^{-1}$ | | 3.3x10$^{-2}$ s$^{-1}$ |
| 29 | XIIa + XII ↔ XIIa = XII → XIIa + XIIa | 1.0x10$^{8}$ M$^{-1}$s$^{-1}$ | 750 s$^{-1}$ | 40 s$^{-1}$ |
| 30 | XIIa + PK ↔ XIIa = PK → XIIa + K | 1.0x10$^{8}$ M$^{-1}$s$^{-1}$ | 3.6x10$^{3}$ s$^{-1}$ | 5.7 s$^{-1}$ |
| 31 | XII + K ↔ XII = K → XIIa + K | 1.0x10$^{8}$ M$^{-1}$s$^{-1}$ | 45.3 s$^{-1}$ | |
| 32 | PK + K → K + K | 2.7x10$^{4}$ M$^{-1}$s$^{-1}$ | | |
| 33 | K → K.inhibited | 1.1x10$^{-2}$ s$^{-1}$ | | |
| 34 | XIIa + C1inh → XIIa = C1inh | 3.6x10$^{3}$ M$^{-1}$s$^{-1}$ | | |
| 35 | XIIa + ATIII → XIIa = ATIII | 21.6 M$^{-1}$s$^{-1}$ | | |
| 36 | XIIa + XI ↔ XIIa = XI → XIIa + XIa | 7.0x10$^{8}$ M$^{-1}$s$^{-1}$ | 200 s$^{-1}$ | 2x10$^{-4}$ s$^{-1}$ |
| 37 | XIa + XI ↔ XIa = XI→ XIa + XIa | 0.7975x10$^{6}$ M$^{-1}$s$^{-1}$ | | |
| 38 | XIa + ATIII → XIa = ATIII | 3.2x10$^{2}$ M$^{-1}$s$^{-1}$ | | |
| 39 | XIa + C1inh → XIa = C1inh | 1.8x10$^{3}$ M$^{-1}$s$^{-1}$ | | |
| 40 | XIa + α1AT → XIa = α1AT | 1.0x10$^{2}$ M$^{-1}$s$^{-1}$ | | |
| 41 | XIa + α2AP → XIa = α2AP | 4.3x10$^{3}$ M$^{-1}$s$^{-1}$ | | |
| 42 | XIa + IX → XIa + IXa | 1.0x10$^{8}$ M$^{-1}$s$^{-1}$ | 41 s$^{-1}$ | 7.7 s$^{-1}$ |
| 43 | IXa + X ↔ IXa = X → IXa + Xa | 2x10$^{-6}$ M | | 6.67x10$^{-4}$ s$^{-1}$ |
| 44 | Xa + VIII ↔ Xa = VIII → Xa + VIIIa | 1.0x10$^{8}$ M$^{-1}$s$^{-1}$ | 20.1 s$^{-1}$ | 0.023 s$^{-1}$ |

**Table 4. Modified Ext model.**

| # | Reaction | $k_1$ | $k_{-1}$ | $k_{cat}$ |
|---|---|---|---|---|
| 1 | TF + VII ↔ TF = VII | $3.2x10^{6}$ $M^{-1}s^{-1}$ | $3.1x10^{-3}$ $s^{-1}$ | |
| 2 | TF + VIIa ↔ TF = VIIa | $2.3x10^{7}$ $M^{-1}s^{-1}$ | $3.1x10^{-3}$ $s^{-1}$ | |
| 3 | TF = VIIa + VII → TF = VIIa + VIIa | $4.4x10^{5}$ $M^{-1}s^{-1}$ | | |
| 4 | Xa + VII → Xa + VIIa | $1.3x10^{7}$ $M^{-1}s^{-1}$ | | |
| 5 | IIa + VII → IIa + VIIa | $2.3x10^{4}$ $M^{-1}s^{-1}$ | | |
| 6 | TF = VIIa + X ↔ TF = VIIa = X → TF = VIIa = Xa | $2.5x10^{7}$ $M^{-1}s^{-1}$ | 1.05 $s^{-1}$ | 6 $s^{-1}$ |
| 7 | TF = VIIa + Xa ↔ TF = VIIa = Xa | $2.2x10^{7}$ $M^{-1}s^{-1}$ | 19 $s^{-1}$ | |
| 8 | TF = VIIa + IX ↔ TF = VIIa = IX → TF = VIIa + IXa | $1.0x10^{7}$ $M^{-1}s^{-1}$ | 2.4 $s^{-1}$ | 1.8 $s^{-1}$ |
| 9 | II + Xa → IIa + Xa | $7.5x10^{3}$ $M^{-1}s^{-1}$ | | |
| 10 | IIa + VIII → IIa + VIIIa | $2.0x10^{7}$ $M^{-1}s^{-1}$ | | |
| 11 | VIIIa + IXa ↔ IXa = VIIIa | $1.0x10^{7}$ $M^{-1}s^{-1}$ | $5.0x10^{-3}$ $s^{-1}$ | |
| 12 | IXa = VIIIa + X ↔ IXa = VIIIa = X → IXa = VIIIa + Xa | $1.0x10^{8}$ $M^{-1}s^{-1}$ | $1.0x10^{-3}$ $s^{-1}$ | 8.2 $s^{-1}$ |
| 13 | VIIIa ↔ $VIIIa_1$ + $VIIIa_2$ | $6.0x10^{-3}$ $s^{-1}$ | $2.2x10^{4}$ $M^{-1}s^{-1}$ | |
| 14 | IXa = VIIIa = X → IXa + X + $VIIIa_1$ + $VIIIa_2$ | $1.0x10^{-3}$ $s^{-1}$ | | |
| 15 | IXa = VIIIa → IXa + $VIIIa_1$ + $VIIIa_2$ | $1.0x10^{-3}$ $s^{-1}$ | | |
| 16 | IIa + V → IIa + Va | $2.0x10^{7}$ $M^{-1}s^{-1}$ | | |
| 17 | Xa + Va ↔ Xa = Va | $4.0x10^{8}$ $M^{-1}s^{-1}$ | 0.2 $s^{-1}$ | |
| 18 | Xa = Va + II ↔ Xa = Va = II → Xa = Va + mIIa | $1.0x10^{8}$ $M^{-1}s^{-1}$ | 103 $s^{-1}$ | 63.5 $s^{-1}$ |
| 19 | Xa = Va + mIIa → Xa = Va + IIa | $1.5x10^{7}$ $M^{-1}s^{-1}$ | | |
| 20 | Xa + TFPI ↔ Xa = TFPI | $9.0x10^{5}$ $M^{-1}s^{-1}$ | $3.6x10^{-4}$ $s^{-1}$ | |
| 21 | TF = VIIa = Xa + TFPI ↔ TF = VIIa = Xa = TFPI | $3.2x10^{8}$ $M^{-1}s^{-1}$ | $1.1x10^{-4}$ $s^{-1}$ | |
| 22 | TF = VIIa + Xa = TFPI → TF = VIIa = Xa + TFPI | $5.0x10^{7}$ $M^{-1}s^{-1}$ | | |
| 23 | Xa + ATIII → Xa = ATIII | $1.5x10^{3}$ $M^{-1}s^{-1}$ | | |
| 24 | mIIa + ATIII → mIIa = ATIII | $7.1x10^{3}$ $M^{-1}s^{-1}$ | | |
| 25 | IXa + ATIII → IXa = ATIII | $4.9x10^{2}$ $M^{-1}s^{-1}$ | | |
| 26 | IIa + ATIII → IIa = ATIII | $7.1x10^{3}$ $M^{-1}s^{-1}$ | | |
| 27 | TF = VIIa + ATIII → TF = VIIa = ATIII | $2.3x10^{2}$ $M^{-1}s^{-1}$ | | |
| 28 | IXa + X → IXa + Xa | $K_m$ = $1,4x10^{-7}$ M | | $K_{cat}$ = 0,048 $s^{-1}$ |
