# Supplementary material for: Critical evaluation of kinetic schemes for coagulation
Source: PLoS One. 2023 Aug 28;18(8):e0290531. doi: 10.1371/journal.pone.0290531 (PMC10461854; doi:10.1371/journal.pone.0290531)
Supplement: Attachment — Submitted filename: Rebuttal_letter.pdf [file pone.0290531.s003.pdf]

Reply to the Reviewer's comments

Alexandre Ranc, Salome Bru, Simon Mendez, Muriel Giansily-Blaizot, Franck Nicoud, and Rodrigo Méndez Rojano

(Dated: April 30, 2023)

Dear Editor, dear Reviewer,

We have carefully read your diligent comments about our manuscript and we have done our best to address them. In the following, we detail a point-by-point answer to the reviewers' comments, reported in blue text.

I. REVIEWER 1

Dear Editor of the PLOS ONE Journal

The manuscript entitled "Critical evaluation of kinetic schemes for coagulation" is an interesting paper because the authors, in addition to presenting the problem in this area, have well illustrated its solution, both in the internal and external coagulation pathways regarding the thrombin generation and thus it certainly deserves publication into the journal, But to meet the increasingly high-quality standard of the Journal some minor revision is needed according to the following points. Furthermore, the authors should consider some grammatical errors correction.

We would like to thank the reviewer for the kind comments. The comments of the reviewer have helped us make the manuscript better. The answers to the comments and suggestions are detailed hereafter.

Comments

1. Using a phrase like "hemophilia A patient" stigmatizes the patient and should be used as " patient with hemophilia A" to maintain the patient's respect.

Expressions like "hemophilia A patient" were modified.

2. "inhouse" OR "in-house". Please use a single form of the word in the text.

"in-house" is now used throughout the text.

3. The sentence "%, and (iv) the lag time (τlag) corresponding to the time required for the generation of 10 nM of thrombin" is not clear. Please rewrite the sentence and specify the role of "%" and determine the expanded form of (iv).

The sentense was rewritten as "...(iii) the time to peak (τ_{max}) which is the time required to reach IIa_{max}, and (iv) the lag time (τ_{lag}) required for the generation of 10 nM of thrombin"

4. The word abbreviations should be expanded in all figures and tables.

Done

5. In the vertical axis of Figure 1, unit (M) is used for unitization. But at the same time, nM is also displayed in this axis, the reason for which is not clear. In addition, the number 10-7 is also mentioned in its upper part, which carries a similar situation. Authors should clarify this.

Figure 1 was modified accordingly to the reviewer's comments.

6. The reason for using Ext and Ent is explained in lines 130 and 131, while lines 65 and 67 refer to these words. Please move this sentence to its original place.

The end of the introduction (lines 64-69) has been rewritten to avoid using the Ext and Int symbols before they are properly defined.

7. in this sentence "Two coagulation kinetic models were used in the current

study. For the sake of simplicity, the models of Chatterjee et al. [2] and Butenas et al. [3] are named Int and Ext, respectively, making reference to the intrinsic and extrinsic pathway." The word "pathway" should be used in plural.

"pathways" is now used instead of "pathway"

8. In line 156 "The rationale for each specific modification is discussed in Section" (And the same in table 1). Section not defined

Section numbers are now correctly indicated.

9. In lines 160 and 178, after IIamax, insert "and" and in line 356, "thombin" should be corrected as "thrombin"

The proposed changes have been made in the revised manuscript.

10. The discussion section should provide more details using the results of the study compared to other research

A wider review of numerical models in literature has been made. We have included a small discussion regarding the work of Pisaryuk et al. [1] in which the original Hockin's model is used to develop a numerical tool to assess individual pharmacokinetic profiles of anticoagulant therapy. They show that thrombin assay simulations differ from the experimental data in the clotting onset regarding the lag time and the amplitude of the thrombin peak as we have. The authors assumed that these discrepancies are minimal as the parameters differ by no more than 15%. An interesting study could be to compute the simulations of Pisaryuk et al. [1] with the proposed modified model. In addition, a paragraph has been added at the beginning of the discussion pointing the reader to a recent review by Chung et al. [2] that offers a more comprehensive look into numerical coagulation models.

11. Some of references are not arranged according to journal guidelines. They

also are not arranged in the same manner. The author should review and correct all of them.

The references have been reviewed and corrected/completed.

II. REVIEWER 2

The authors compared the intrinsic and extrinsic pathways of coagulation with thrombin generation assays considering realistic pathological conditions. In my opinion, this manuscript can be accepted after applying the requested amendments.

We would like to thank the reviewer for the report and comments that helped us simplify and improve our manuscript. The details of changes are detailed below.

Comment 1: -The abstract is somewhat unintelligible; it is hard to understand what are the aim, methods, results and conclusion of the study.

The abstract has been rewritten and now reads as follows:

Two well-established numerical representations of the coagulation cascade either initiated by the intrinsic system (Chatterjee et al., PLOS Computational Biology 2010) or the extrinsic system (Butenas et al., Journal of Biological Chemistry, 2004) were compared with thrombin generation assays under realistic pathological conditions. Biochemical modifications such as the omission of reactions not relevant to the case studied, the modification of reactions related to factor XI activation and auto-activation, the adaptation of initial conditions to the thrombin assay system, and the adjustment of some of the model parameters were necessary to align in vitro and in silico data. The modified models are able to reproduce thrombin generation for a range of factor XII, XI, and VIII deficiencies, with the coagulation cascade initiated either extrinsically or intrinsically. The results emphasize that when existing models are extrapolated to experimental parameters for which they have not been calibrated, careful

adjustments are required.

Comment 2: -In general, the article is written complex and should be written in a simpler and more understandable way. For example, page 1, lines 11, 12, 13 Numerical representations of the coagulation cascade aim to mimic the thrombin generation process which is the result of the balance between prothrombin conversion and thrombin inactivation [9], thrombin being the key enzyme of the blood clotting cascade.

The sentence has been simplified into: "Numerical representations of the coagulation cascade aim to reproduce the thrombin generation process, which results from the balance between prothrombin conversion and thrombin inactivation."

Long sentences of the original manuscript have been cut or simplified in the revised version.

Comment 3: -Using a combination of different words for the same concept will be confusing; it is suggested to use the same words. For example, Numerical representations numerical modeling Numerical data numerical cases numerical thrombin production ...

The wording was condensed to numerical models and simulation results.

Comment 4: -What does (. %,) mean in the following line? If it is wrong, correct it. page 4, line 125 ... (iii) the time to peak (τ_{max}) which is the time required to reach IIamax [20]. %, and (iv) the...

The typo has been corrected in the revised manuscript.

Comment 5: page 13, line 325 As pointed out in [25, 26] the values of... -I

suggest that the names of the authors be used.

The names of the authors have been added in the text.

Comment 6: -The first paragraph of the discussion section is suitable for the introduction. It is better to merge this part with the introduction. This change will lead to a better understanding of the purpose of the study.

We thank the reviewer for this suggestion. This paragraph was merged with the introduction.

III. REVIEWER 3

Despite the fact that numerical modeling of the coagulation cascade has a long history at the moment there are gaps in this area of research. The results of numerical modeling should be the study of the pharmacokinetics of new drugs and the study of the dynamics of thrombosis in pathological conditions. Well-validated quantitative models of the coagulation cascade are expected to complement traditional laboratory as predictive tools in clinical practice, enabling physicians to estimate disease risk or simulate therapeutic outcomes in individual patients. In this paper, the authors evaluated two well-established mathematical models of the coagulation cascade for the contact pathway and for the TF path in conditions of clotting factor VIII, XI or XII deficiencies. The authors describe the modifications made for the Int and Ext models. The proposed modifications to the Int and Ext models can be used as a valuable tool to explore any scenario in a less expensive way compared to the experimental path. Given the complexity kinetic schemes of the coagulation cascade, the authors call for close cooperation between hematologists and modelers in the application of coagulation models. The article has a traditional structure. A sufficient number of tables and figures make it easier to understand the work done. The list of references is represented by a large number of publications from the period

from 1990 to 2021.

We would like to thank the reviewer for the careful reading of our paper, the accurate report and positive comments.

[1] A. S. Pisaryuk, N. M. Povalyaev, A. V. Poletaev, and A. M. Shibeko, Systems Biology Approach for Personalized Hemostasis Correction, Journal of Personalized Medicine **12**, 10.3390/jpm12111903 (2022).

[2] D. Chung, S. Bakshi, and P. H. van der Graaf, A Review of Quantitative Systems Pharmacology Models of the Coagulation Cascade: Opportunities for Improved Usability, Pharmaceutics **15**, 918 (2023).
