# Supplementary material for: Critical evaluation of kinetic schemes for coagulation
Source: PLoS One. 2023 Aug 28;18(8):e0290531. doi: 10.1371/journal.pone.0290531 (PMC10461854; doi:10.1371/journal.pone.0290531)
Supplement: Attachment — Submitted filename: Dear Editor of the PLOS ONE Journal.docx [file pone.0290531.s002.docx]

**Dear Editor of the** PLOS ONE **Journal**

The manuscript entitled “Critical evaluation of kinetic schemes for coagulation” is an interesting paper because the authors, in addition to presenting the problem in this area, have well illustrated its solution, both in the internal and external coagulation pathways regarding the thrombin generation and thus it certainly deserves publication into the journal, But to meet the increasingly high-quality standard of the Journal some minor revision is needed according to the following points. Furthermore, the authors should consider some grammatical errors correction.

**Comments**

1. Using a phrase like "hemophilia A patient" stigmatizes the patient and should be used as " patient with hemophilia A" to maintain the patient's respect.

2. “inhouse” OR “in-house”. Please use a single form of the word in the text.

3. The sentence “%, and (iv) the lag time (τlag) corresponding to the time required for the generation of 10 nM of thrombin” is not clear. Please rewrite the sentence and specify the role of "%" and determine the expanded form of (iv).

4. The word abbreviations should be expanded in all figures and tables.

5. In the vertical axis of Figure 1, unit (M) is used for unitization. But at the same time, nM is also displayed in this axis, the reason for which is not clear. In addition, the number 10^-7^ is also mentioned in its upper part, which carries a similar situation. Authors should clarify this.

6. The reason for using Ext and Ent is explained in lines 130 and 131, while lines 65 and 67 refer to these words. Please move this sentence to its original place.

7. in this sentence "Two coagulation kinetic models were used in the current study. For the sake of simplicity, the models of Chatterjee et al. [2] and Butenas et al. [3] are named Int and Ext, respectively, making reference to the intrinsic and extrinsic pathway." The word "pathway" should be used in plural.

8. In line 156 “The rationale for each specific modification is discussed in Section” (And the same in table 1). Section not defined

9. In lines 160 and 178, after IIamax, insert “and” and in line 356, “thombin” should be corrected as “thrombin”

10. The discussion section should provide more details using the results of the study compared to other research

11. Some of references are not arranged according to journal guidelines. They also are not arranged in the same manner. The author should review and correct all of them.
